# ATR-FTIR-MIR Spectrometry and Pattern Recognition of Bioactive Volatiles in Oily versus Microencapsulated Food Supplements: Authenticity, Quality, and Stability

**DOI:** 10.3390/molecules26164837

**Published:** 2021-08-10

**Authors:** Ramona Maria Popa, Florinela Fetea, Carmen Socaciu

**Affiliations:** 1 Department of Food Science, Faculty of Food Science and Technology, University of Agricultural Sciences and Veterinary Medicine Cluj-Napoca, 400372 Cluj-Napoca, Romania; ramonapopa2402@gmail.com (R.M.P.); florinelafetea@usamvcluj.ro (F.F.); 2 Research Center for Applied Biotechnology, Proplanta, 400478 Cluj-Napoca, Romania

**Keywords:** Fourier transform infrared spectroscopy, essential oils, microencapsulation, authenticity, quality, stability, food supplements

## Abstract

Fourier transform infrared spectroscopy on the middle infrared region (ATR-FTIR-MIR) proved to be a convenient and reliable technique to evaluate foods’ quality and authenticity. Plants’ essential oils are bioactive mixtures used as such or in different oily or microencapsulated formulations, beneficial to human health. Six essential oils (thyme, oregano, juniperus, tea tree, clove, and cinnamon) were introduced in three oily formulations (Biomicin, Biomicin Forte, and Biomicin urinary) and these formulations were microencapsulated on fructose and maltodextrin matrices. To study their stability, the microencapsulated powders were kept under light irradiation for 14 days at 25 °C or introduced in biopolymer capsules. All variants were analysed by ATR-FTIR-MIR, recording wavenumbers and peak intensities (3600–650 cm^−1^). The data were processed by Unscrambler and Metaboanalyst software, with specific algorithms (PCA, PLSDA, heatmaps, and random forest analysis). The results demonstrated that ATR-FTIR-MIR can be successfully applied for fingerprinting and finding essential oil biomarkers as well as to recognize this pattern in final microencapsulated food supplements. This study offers an improved ATR-FTIR-MIR procedure coupled with an adequate chemometric analysis and accurate data interpretation, to be applied for the evaluation of authenticity, quality, traceability, and stability during storage of essential oils incorporated in different matrices.

## 1. Introduction

Essential oils (EOs), also known as volatile oils, contain mainly components that evaporate easily, namely, hydrocarbons like mono- and diterpenes, terpenoid derivatives (alcohols, aldehydes and ketones, esters, and ethers) accounting for 90–98%, as well as some less volatile components. Dozens of articles have been reported in recent years on the chemical composition of volatiles from aromatic plants, e.g., spontaneous flora or cultivated flora in continental, tropical, and subtropical areas. Their composition is greatly influenced by genotype (species, cultivation, and ecotype), ecological (geographical origin, climate, and soil composition), and technological factors (cultivation, storage of raw material, and processing techniques) [1]. EOs are widely used as natural flavors of food, being responsible for a good taste and aroma. Important sources of EOs are found in culinary herbs from the *Lamiaceae* family (thyme, sage, oregano, peppermint, marjoram, and basil), in coniferous (juniperus and pine), as well in spices (clove and cinnamon).

The addition of herbs and spices, as well as EOs, are natural solutions for the protection of fatty foods, as it can control rancidity, delay the formation of toxic oxidation products, maintain nutritional quality, and extend the shelf life of food products [2]. Meanwhile, cheaper, synthetic variants, which falsify EOs, are found in the market; therefore, their authentication and traceability is an important area of investigation [3].

EOs from aromatic plants are highly appreciated by consumers nowadays, owing to their antioxidant effects and free radical scavengers [4,5]. Moreover, as demonstrated in recent decades, through various in vitro or in vivo experiments, EOs act as antimicrobials, protecting against respiratory, infectious, and cardiovascular diseases [6,7]. Many components of EOs were identified as effective antibiotics, e.g., carvacrol, thymol, eugenol, cinnamaldehyde, and cinnamic acid.

Thyme EOs contain mainly volatiles like thymol and terpinene and have anti-inflammatory, antimicrobial, and antioxidant activities, related mainly to thymol content [8,9,10,11,12]. Oregano contains approximately 70% thymol and carvacrol, dependent on geographic origin and plant developing stage [13,14], and shows anti-inflammatory, antispasmodic, antifungal, and antibacterial activity [15]. Juniperus is rich in monoterpenes (approximately 58%), mainly α-pinene, β-myrcene, and sabinene, and has antimicrobial activity and industrial applications [16,17].

Tea tree, originating from Australia, is used for its essential oil rich in monoterpenes and sesquiterpenes, mainly methyl eugenol. Its oil has high commercial value for the pharmaceutical and cosmetic industry. Its composition was characterized by gas chromatography coupled with mass spectrometry (GC-MS) and spectroscopy [10,18,19]. Tea tree essential oil showed antiseptic, cytotoxic, antifungal, antiviral, and anti-inflammatory activity [18,19], including a high potency against multi drug-resistant bacteria [20].

Clove, of Indonesian origin, is being cultivated nowadays in many regions as a rich source of eugenol and eugenol acetate. It has high potential for pharmaceutical, cosmetic, and food applications thanks to its antioxidant and antimicrobial effects [21,22,23,24,25].

The bark of Cinnamon, cultivated in South East Asia, is utilized in food, pharmaceuticals, and cosmetics. Its essential oil includes mainly cinnamaldehyde (up to 88%), but also coumarin, cinnamic alcohol, cinnamic acid, and 2-metoxicinnamaldehyde. Its biological properties (antioxidant, antidiabetic, thrombocyte anti-aggregation, antifungal, and antibacterial) were recently described [10,26,27,28,29].

As they are complex mixtures with diverse and intense antimicrobial activities, these EOs are an interesting alternative to antibiotics and are indicated in treatments against antibiotic-resistant bacteria. In vitro studies have shown their antibacterial activity especially against pathogenic Gram-negative bacteria, such as *Listeria*, *Salmonella*, *Escherichia coli*, *Shigella*, and *Staphylococcus aureus*, as well against fungi and yeasts [30].

For food and cosmetic applications, EOs are usually dissolved in edible vegetable oils, such as sunflower oil, olive oil, coconut oil, and so on. Such edible oils act as stabilizers, being rich in unsaturated fatty acids, tocopherols, and carotenoids, having an antioxidant effect.

New formulations were obtained by Eos’ microencapsulation in different matrices (solid, inert, nontoxic micron-size particles, or hydrocolloids). Various strategies and techniques are currently applied, either for EOs or their oily mixtures. The adsorption on the matrix surface stabilizes the volatile components against inadequate temperature, humidity, and oxygen [31]. Microencapsulation became a common technique for obtaining pharmaceuticals and dietary supplements, also being used for targeted medical therapies [32,33,34,35].

Spray drying (atomization) is widely used for encapsulating EOs on low-cost matrices, e.g., maltodextrin and starch [36,37]. Coacervation uses arabic gum, gelatin, chitosan, or xanthan as matrices and glutaraldehyde as the crosslinking agent, e.g., *Citronella* essential oil was encapsulated with an efficiency of approximately 94% [38,39]. Nanoliposomes were also good vehicles for volatiles’ encapsulation [40]. Commonly used matrices are also plant-derived or algae carbohydrates (modified starch and maltodextrins, inulin, fructose and alginates, and carrageenan, respectively [41,42]), as well as whey proteins and protein–carbohydrate complexes [43,44,45,46] or nanofibers [25]. Such matrices were also used to retain volatile, toxic environmental hydrocarbons [47].

The powders obtained by microencapsulation can be used as such or introduced in gelatin/polymer capsules in chewable pills. Maltodextrin is a carbohydrate derivative obtained by partial enzymatic hydrolysis of starch and has a lower glycemic index. It is used to improve the taste of chips and energy drinks as a dietary supplement for athletes [48]. It allows a good retention of volatile compounds [49], while fructose, like sucrose or inulin, has a high retention capacity and attractive sensory qualities [32].

The routine characterization of essential oils is performed in quality control laboratories of manufacturing companies by their physical parameters included in technical specifications. These are density, refractive index, solubility in alcohol, freezing point, boiling point, rotational power, and peroxide index. In highly specialized laboratories, EOs and their different formulations can be investigated by high resolution techniques, such as GC-MS, but also by fast and non-destructive methods, such as Fourier transform infrared spectrometry (FTIR) or Raman spectrometry [50]. These are validated by comparison with GC-MS. Over the last few decades, attenuated total reflectance-Fourier transform infrared (ATR-FTIR) spectroscopy has become an attractive alternative to traditional methods, being a rapid and easy-to-use technique to evaluate liquid samples. Therefore, EOs’ edibility, intrinsic quality parameters, safety, as well as authenticity and traceability can be achieved by ATR-FTIR [51,52,53]. Nevertheless, FTIR spectra are complex, including overlapped vibrations of molecules and various vibrational modes. Such complexity makes the interpretation very difficult [54].

The spectral range from 4000 to 400 cm^−1^ (middle infrared region—MIR) is optimal for the identification of organic groups with characteristic vibrations. Moreover, the quantitative evaluation of components is possible if adequate calibrations are complemented [55].

FTIR-MIR spectroscopy is crucially dependent on the chemometric methods, including calibrations with large and representative sample numbers [56]. Common qualitative unsupervised approaches include principal component analysis (PCA), cluster analysis (CA), or soft independent modeling of class analogy (SIMCA), while supervised methods include partial least squares discriminant analysis (PLSDA) and linear discriminating analysis, among others [57]. The random forest algorithm is applied as a one-class classifier and shows superior performance for classification and authentication [58]. The web-based software MetaboAnalyst 5.0 (https://www.metaboanalyst.ca/, accessed on 10 July 2021) is a complex biostatistical tool that accepts a variety of input data (from MS to FTIR peak lists). It offers many options for data processing, from univariate (analysis of variance (ANOVA) *t*-test) to multivariate statistical analysis, as well other sophisticated statistical or machine learning methods.

Different reviews and experimental data have been reported about FTIR spectroscopy applied especially to edible oils to check their quality, authenticity, as well detection of adulteration [59,60,61], or to assess antioxidant activity [62]. Compared with data available about edible oils, fewer data were reported for the characterization of essential oils and derived products. Our group applied ATR-FTIR-MIR spectrometry for different liquid foods (beverages, fruit juices, or edible oils), in parallel with calibrations using GC-MS or LC-MS techniques for food authentication and quality assessment [63,64].

Recently, we established the comparative GC-MS signatures of six EOs used as food flavors [65]. To complement this study, here, we applied ATR-FTIR-MIR spectrometry to find the pattern of the same six EOs incorporated in different formulations (oily and microencapsulated on fructose and maltodextrin). The stability of Eos’ components in solid formulations was investigated before and after light irradiation and storage in the dark of biopolymer capsules. All data were processed using Unscrambler X.10.4 or Metaboanalyst 5.0 software. The goals of this investigation are multiple: recognition of Eos’ main components in these formulations; tracing the specific biomarkers in oily and microencapsulated products; and checking their quality, authenticity, and stability.

## 2. Results

### 2.1. ATR-FTIR-MIR Spectra of the Oily Formulations

**Figure 1:**
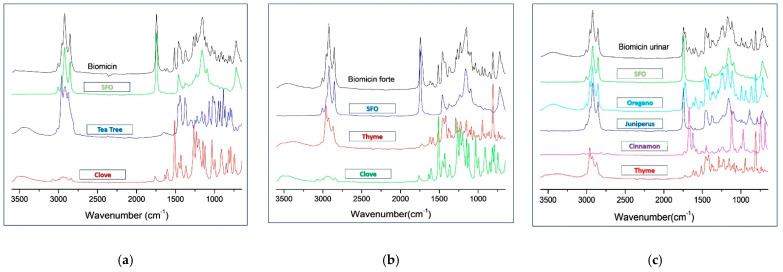
Comparative ATR-FTIR-MIR spectra (3600–650 cm^−1^) for Biomicin (**a**), Biomicin Forte (**b**), and Biomicin urinary (**c**), compared with SFO and EOs from the plant ingredients.

Figure 1a–c comparatively represents the ATR-FTIR-MIR spectra (3600–650 cm^−1^) for Biomicin, Biomicin Forte, and Biomicin urinary, in parallel with spectra of SFO and Eos’ ingredients (mentioned in the figure).

Tables S1–S3 include the wavenumbers (cm^−1^) corresponding to the main peaks identified in the ATR-FTIR-MIR spectra of Biomicin (Table S1), Biomicin forte (Table S2), and Biomicin urinary (Table S3) compared with sunflower oil (SFO).

The contribution of each EO in the oily mixtures can be seen qualitatively in spectra. The specific peaks of Biomicin show, compared with SFO, that the contribution of clove is seen in the regions 1255–1265, 1510–1512, and 1591–1606 cm^−1^; the contribution of tea tree in the region 2900–3010 cm^−1^; and the contribution of both clove and tea tree in the regions 744–785, 792–810, 993–997, and 1091 cm^−1^, as shown in Figure 1a and Table S1. Similarly, the contribution of clove in Biomicin Forte is seen in the regions 1056–1058, 1261, and 1510–1514; that of both thyme and clove at 1583–1618 cm^−1^; and that of thyme at 2956 cm^−1^ (Figure 1b). As can also be seen in Table S2, clove has an important influence by its specific absorptions at 1056, 1288, and 1583 cm^−1^. Biomicin urinary has a more complex composition; therefore, the differences between the product and SFO fingerprints are more significant, especially in the regions 744–810, 1255–1301 cm^−1^ (specific to thyme and oregano), 941 and 1740 cm^−1^ (specific to juniper), 993 cm^−1^ (specific to oregano), 1172 and 1502–1622 cm^−1^ (specific to cinnamon), and 1724 and 2956 (thyme, oregano and juniper), as can also be seen in Figure 1c and Table S3.

**Figure 2:**
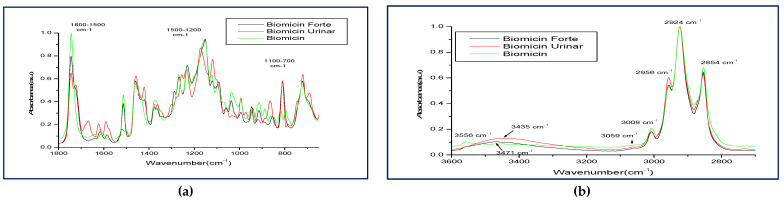
Comparative ATR-FTIR-MIR spectra registered in two regions: 1800–650cm^−1^ (**a**) and 3600–2700 cm^−1^ (**b**), for Biomicin, Biomicin Forte, and Biomicin urinary, to show the wavenumbers and peak intensities that may differentiate these products.

Based on these observations, Figure 2 shows the superposed ATR-FTIR-MIR fingerprints of Biomicin, Biomicin Forte, and Biomicin urinary, as registered in regions 1800–650 cm^−1^ (a) and 3600–2700 cm^−1^ (b).

Different peak shapes and intensities can discriminate the three formulations, visible especially in the regions 817–862, 1420–1460, 1560–1743 cm^−1^ (Figure 2a), as well as 2956–3435 cm^−1^ (Figure 2b) For a betrer, semiquantitative evaluation, the peak intensities for each product are presented in Table 1. The significant recognition markers expressed by wavenumbers (WNs) and their intensities are included in frames, as seen below.

As a semi-quantitative evaluation, one can discriminate each of these three products: Biomicin with high intensities at 744, 796, 817, and 1199 cm^−1^. Biomicin and Biomicin forte showed similar WNs, but different intensities, while Biomicin urinary had specific WNs at 744, 785, 972, 1301, 1361, 1502, 1622, 1668, and 1724 cm^−1^.

### 2.2. Comparative FTIR Spectra of Different Microencapsulated Products

**Figure 3:**
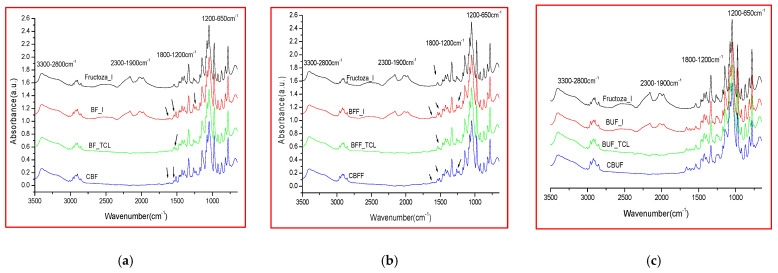
FTIR spectra (3500–650 cm^1^) of microencapsulated products Biomicin (**a**), Biomicin forte (**b**), and Biomicin urnary (**c**) using fructose as matrix, at initial stage (I), after light irradiation at 25 °C (TCL), and in biopolymer capsules (C). For abbreviations, see *Materials and Methods*.

**Figure 4:**
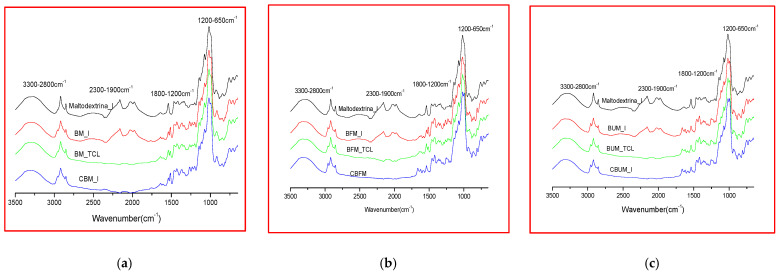
FTIR spectra (3500–650 cm^1^) of microencapsulated products Biomicin (**a**), Biomicin forte (**b**), and Biomicin urinary (**c**) using maltodextrin as matrix, at initial stage (I), after light irradiation at 25 °C (TCL), and in biopolymer capsules (C). For abbreviations, see *Materials and Methods*.

Two categories of microencapsulated products were obtained from each oily formulation (Biomicin, Biomicin Forte, and Biomicin Urinary); the first one using fructose as a matrix and the second one using maltodextrin. Comparative spectra were registered for each sample at initial stage (I), after light irradiation at room temperature (TCL) and after inclusion in biopolymer capsules (C) (Figure 3 and Figure 4, respectively). For abbreviations, see Materials and Methods. Figure S1 shows the superposed ATR-FTIR-MIR spectra (3500–650 cm^−1^) of matrices fructose (a) and maltodextrin (b) at initial stage (I) and after daylight irradiation over 14 days at 25 °C (TCL). Tables S4–S9 include the wavenumbers and peak intensities for each experimental variant.

Interestingly, at the initial state (I), the spectra of matrices and samples showed absorption peaks that are no longer found after storage in the light or in the capsule. These signals found at 1975, 2027–2029, and 2160–2166 cm^−1^ are in agreement with literature data showing that the infrared region at 1900–2300 cm^−1^ is attributed to carbon dioxide bonds [66]. Compared with oily formulations, in the spectra of the microencapsulated products, the absorption intensities in the region 3500–2700 cm^−1^ are significantly reduced compared with intensities in the region 1600–650 cm^−1^.

### 2.3. Multivariate Analysis of Microencapsulated Products

**Figure 5:**
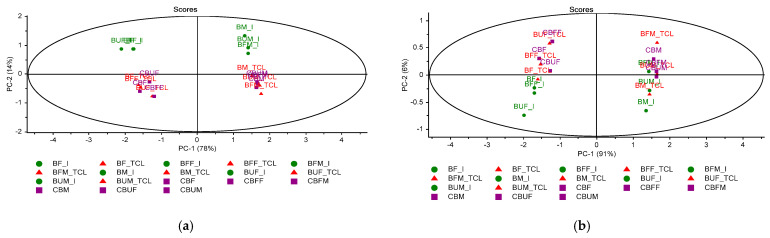
Principal component analysis (PCA) score plots corresponding to the IR region 3600–650 cm^−1^ (**a**) and 1800–650 cm^−1^ (**b**) including the comparative fingerprints of microencapsulated products Biomicin (B), Biomicin Forte (BF), and Biomicin urinary (BU) using fructose (F) vs. maltodextrin (M) as matrices, at initial stage (I), after light irradiation at room temperature (TCL), and after inclusion in biopolymer capsules (C). For abbreviations, see *Materials and Methods*.

Using the Unscrambler algorithms, PCA score graphs were built as presented in Figure 5.

The PCA score plot for the whole MIR region 3600–650 cm^−1^ showed a good discrimination (co-variance of 92%) between products at the initial stage (I) and products kept for 14 days in light at 25 °C (TCL) or in capsules (C) (Figure 5a). One main reason of differentiation is the presence of CO_2_ peaks at the initial stage (I). Looking to the fingerprint region 1800–650 cm^−1^ (Figure 5b), a larger distribution was noticed, with the co-variance being 97%. Therefore, a good discrimination was observed between products made on fructose and maltodextrin, as well between the initial to final stages (TCL or C).

### 2.4. One-Way ANOVA Univariate Analysis

**Figure 6:**
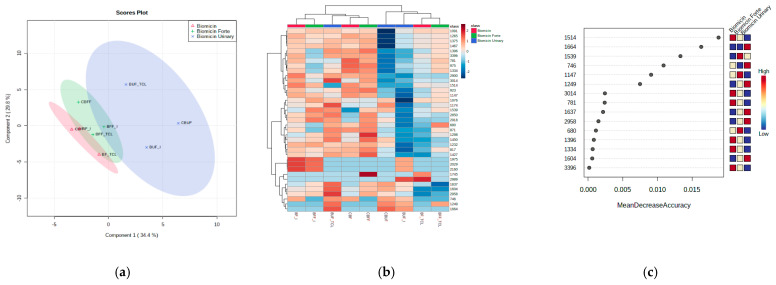
Partial least squares with discriminant analysis (PLSDA) score plot (**a**), heatmap (**b**), and RF ranking analysis (**c**) of microencapsulated products Biomicin, Biomicin forte, and Biomicin urinary using fructose (F) as a matrix, at the initial stage (I), after light irradiation at room temperature (TCL),fand after inclusion in biopolymer capsules (C). For abbreviations, see *Materials and Methods*.

**Figure 7:**
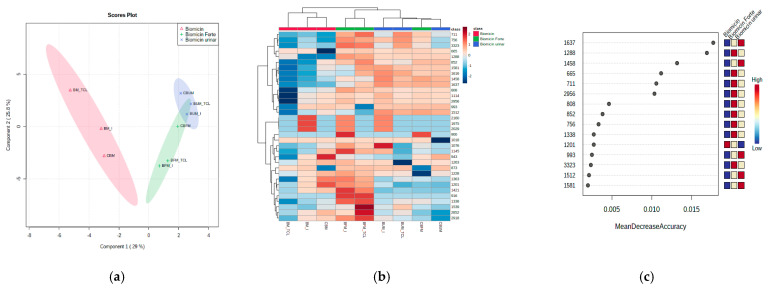
Partial least squares with discriminant analysis (PLSDA) score plot (**a**), heatmap (**b**), and RF ranking analysis (**c**) of microencapsulated products Biomicin, Biomicin Forte, and Biomicin urinary using maltodextrin (M) as a matrix, at the initial stage (I), after light irradiation at room temperature (TCL), and after inclusion in biopolymer capsules (C). For abbreviations, see *Materials and Methods*.

The partial least squares discriminant analysis (PLSDA) score plot coupled with the correlation heatmap and the random forest (RF) analysis were considered for a better discrimination between products. Figure S2 shows the correlation maps between microencapsulated samples on fructose (a) and maltodextrin (b). Figure 6 and Figure 7 show the PLSDA score plots (a), the correlation heatmaps (b), and random forest ranking analysis (c) for Biomicin, Biomicin forte, and Biomicin urinary using fructose (F) and maltodextrin (M), respectively. The following were compared: the initial stage (I) versus light irradiation (TCL) versus inclusion in capsules (C).

The PLSDA score plots (Figure 6a and Figure 7a) showed good discriminations between products (co-variance of 64.2% and 54%) when the matrix was fructose and maltodextrin, respectively. Nevertheless, a stronger delimitation of Biomicin against Biomicin Forte and Biomicin urinary was noticed for maltodextrin-based products. This is also evident in the heatmaps (Figure 6b and Figure 7b), with the differences between samples being due mainly to lower intensities in the region 1600–800 cm^−1^. The heatmaps reflect, in an intuitive manner, the different correlations (colored from positive red to negative blue) between individual samples and their specific WNs. In addition, the clusters of the samples are visible in the upper part of each matrix.

Using a cutoff *p*-value < 0.001 and the mean decrease accuracy (MDA) values >0.01, as shown by RF graphs (Figure 6c and Figure 7c), the most significant wavenumbers were ranked, which may be considered as predictive biomarkers for a good discrimination among the product groups of oils. These are 1514, 1664, 1539, and 746 cm^−1^ for products encapsulated on fructose and 1637, 1288, 1458, 665, 711, and 2956 cm^−1^ for products encapsulated on maltodextrin. Biomicin made on maltodextrin showed significant differentiation at these regions, being especially negatively correlated against Biomicin Forte.

### 2.5. Recognition Patterns and Stability of Microencapsulated Products

Considering the detailed spectral data (WN vs. peak intensity) presented in Tables S4–S9, the specific WNs were identified that can be used to recognize each product (Biomicin, Biomicin Forte, and Biomicin urinary) on specific matrices (fructose and maltodextrin), as well as the modifications induced by light exposure (Table 2).

The data included in Table 2 are in good agreement with the data from Table 1, which referred to the oily formulations. The specific WNs are used to recognize every formulation as well as their ingredients (plant EOs). The products included in capsules remained as stable as in the initial stage. After light irradiation, only slight modifications of peak intensities TCL and initial stage (%TCL/I) were observed. The most significant change was noticed for Biomicin urinary on fructose, where increases of 20–29% of peak intensities were observed at 1589, 1622, and 1664 cm^−1^.

We considered to identify possible oxidations during light exposure, looking to changes in the FTIR spectrum [64], at 722 and 3006 cm^−1^ (corresponding to vibrations of cis double bonds), in the region 1720–1750 cm^−1^ for carbonyl groups C=O, and in the fingerprint region 1500−900 cm^−1^. No modifications were found in any product, thus both microencapsulation matrices successfully stabilized the products Biomicin, Biomicin Forte, and Biomicin urinary. The peaks specific to EOs integrated in these products were still present, without oxidative changes; they have shown a good stability.

## 3. Discussion

The investigations and results presented here demonstrated that ATR-FTIR-MIR can be successfully applied successively to fingerprint plant-derived essential oils, identifying their pattern in oily, as well as in microencapsulated formulations.

The ATR-FTIR-MIR absorption spectra of essential oils show characteristic vibrations like C-H stretch (~2900 cm^−1^), C=O stretch (~1700 cm^−1^), broad O-H stretch (~3400 cm^−1^), and C-O stretch (~1100 cm^−1^) for terpenoids components; therefore, they are dominated by vibrational modes of monoterpenes at 886, 1436, and 1644 cm^−1^. The differential identification of many oils is the band for C=O stretching, at 1740 cm^−1^ in rosemary and sage oil, shifted to lower wavenumbers in oregano (terpineol type). Specific to *Lamiaceae* family group EOs (thyme and oregano) in the FTIR spectra, the strongest influence is due to bands at 842 cm^−1^ in thyme, and 862 cm^−1^ in oregano, respectively, bands from ~1375 and 1450 cm^−1^, and a broad band at 3400–3500 cm^−1^ [54].

As seen in Section 2.1, the contribution of individual essential oils gives specific patterns of the spectra, as shown in Figure 1 for oily formulations, where Biomicin urinary had the most complex composition, and the contribution of individual EOs can be identified. The peak shapes and intensities reflected a good discrimination between the three formulations, as shown in Figure 2a–c and Table 1. The recognition markers for each formulation expressed by wavenumbers (WNs) and peak intensities can be used for a fast screening of these formulations in any laboratory with an FTIR spectrometer.

As presented in Section 2.2, the spectra of the microencapsulated products showed generally reduced absorption intensities in the region 3500–2700 cm^−1^ compared with the region 1600–650 cm^−1^, owing to the absence of sunflower oil. It is known that edible oils have specific lipid absorptions in the region 2953–2854 cm^−1^.

The chemometric analysis was very useful to identify and discriminate differences between the profiles of the three formulations in each of the matrices (fructose or maltodextrin), as shown in Section 2.3, Section 2.4, Section 2.5 and Figure 5, Figure 6 and Figure 7. It offered the possibility to see in an intuitive way the discriminations between samples, between the effect of matrices, and the impact of light irradiation. The PLSDA score plots showed that maltodextrin-based formulations were able to better discriminate the different Eos’ ingredients. A general picture of the comparisons between samples and significant wavenumbers is offered by heatmaps, offering a fast visual approach of differences. The random forest analysis and the accuracy parameter MDA showed which are the most relevant wavenumbers able to discriminate the different EOs adsorbed on these matrices.

The specific absorption wavenumbers and peak intensities before and after light exposure (Table 2) also indicated a good stability of these solid formulations as well the differences between fructose versus maltodextrin. Maltodextrin is recommended for encapsulation, having the advantage of lower hygroscopicity, better stability, and better availability to be included in food (e.g., candies and gums) or food supplements.

Compared with oily formulations, more exposed to oxidation and more difficult to introduce in food matrices, solid microencapsulated formulations, especially on maltodextrin, are recommended.

The contribution of each component (thyme, oregano, juniper, tea tree, clove, and cinnamon) in essential oils, in less volatile oily formulations, and in solid encapsulated formulas was easily identified and evaluated semi-quantitatively by specific FTIR absorption wavenumbers and intensities. These data complemented our previous studies that used the GC-MS technique to separate individual molecules from the same six EOs, finding their major biomarkers of authenticity, e.g., thymol and p-cymene for thyme; α-pinene, β-myrcene, and sabinene for juniper; carvacrol for oregano; terpinene derivatives for tea tree, eugenol and eugenol acetate for clove; and cinnamaldehyde for cinnamon.

Similar studies conducted for essential oils extracted from thyme [9,10,11], oregano [13,14], juniper [16], tea tree [18,19], clove [21,22,23,24], and cinnamon [26,27,28], some of them validated by GC-MS, showed FTIR spectrometry to be a reliable method to evaluate their pattern and key biomarkers. These studies also confirm our findings. None of these were focused on essential oils encapsulated in solid matrices.

Some reviews [51,52,53,55,56] and experimental studies reflected the usefulness of FTIR spectrometry for larger classes of fats and oils, combined with chemometrics. Experimental studies conducted on plant essential oils, beside the ones mentioned above, demonstrated the advantages of FTIR spectroscopy for the quality, authentication, and safety of essential oils [54,56]. Mainly, such studies focused on essential oils’ taxonomy and purity using chemometrics for discrimination analysis [54], for its capability to detect and quantify adulterants, e.g., in lemon essential oil [57] or menthe [60]. Only one recent study, which used pure volatiles and standardized clove and spearmint EOs encapsulated on organic (confidential) matrices, was published, applying GC-MS and chemometric analysis in parallel [23].

GC-MS is an accurate, expensive method dedicated to the identification of individual components in high standard laboratories, useful for quality standardization of free essential oils. Previous studies, including ours, found good correlations between FTIR spectrometric results and GC-MS, with the great advantage of FTIR being its capability to carry out an analysis in a short time, in a non-destructive manner, by simple procedures, and of many samples. Moreover, FTIR analysis can also be conducted on free and liquid or solid formulations directly, without any chemical interventions.

The novelty of our study lies in the simplified ATR-FTIR-MIR protocol for analyses in a short time of a large number of complex mixtures, identifying the specific phenotype of each ingredient in complex mixtures, either as oily formulation or encapsulated on solid matrices. It shows the different patterns of solid formulations, comparing fructose and maltodextrin, in order to find one suitable and more stable against light exposure and oxidation. Fructose was not reported until now to be used as an encapsulation matrix, in spite of its natural origin and high nutritional quality. The advantage of maltodextrin formulation comes from its good taste, stability, and gradual release of the essential oil components, with some publications reflecting its advantages as a useful ingredient for encapsulation, especially when mixed with starch, alginate, or arabic gums [37,41,42].

As free EOs or their formulations proved to be well accepted by scientists and consumers as natural ingredients in food supplements or cosmetics [1,2,7,67], this study may offer an example of a systematic ATR-FTIR-MIR procedure that integrates the pattern recognition of EOs’ fingerprints, a semi-quantitative evaluation coupled with a chemometric analysis, and accurate data interpretation. Such a procedure allows a more accurate evaluation of the authenticity of essential oils, their quality and traceability in derived formulations and final products, as well their stability during storage or inside different matrices.

## 4. Materials and Methods

### 4.1. Materials

#### 4.1.1. EOs and Oily Formulations

A total of six pure EOs, provided from company Fares (Oraştie, Romania) were analyzed: thyme, juniperus, oregano, clove, cinnamon, and tee tree. The GC-MS fingerprinting and semi-quantitative composition were previously reported [65].

A total of three original oily formulations containing combinations of 2–4 EOs dissolved in sunflower oil (see Figure 1) were also produced by Fares company in Romania https://fares.ro/en/, (accessed on 10 July 2021). The brand names of these products, which are yet to be commercialized, are Biomicin, Biomicin Forte, and Biomicin urinary. All samples were previously characterized by various parameters in the company, as mentioned in their standard procedures SOP.

#### 4.1.2. Microencapsulated Powders Obtained from Biomicin Brand Products

Two types of microcapsules were obtained, using fructose (F) and maltodextrin (M). The recipes of microcapsules made on fructose (F) included microcrystalline cellulose, Aerosil, and Mg stearate, as well as the oily products Biomicin (B), Biomicin Forte (BF), and Biocin urinary (BU), while the microcapsules made on maltodextrin (M) included lactose, Aerosil, Mg stearate, and the same three oily products, as presented in Table 3.

Both powders ingredients, fructose and maltodextrin had particle sizes <1 mm. The technological steps to obtain microencapsulated formulas included the following: homogenization of the dry powders in the malaxating machine Cardinal, addition of oily products (Biomicin, Biomicin forte, and Biomicin urinary), and drop-by-drop under mixing over 30 min in a dark room. Three parallel samplings were prepared for each product. Then, microencapsulated powders were dried at 36 °C in an air Convert Dryer and homogenized again in a Cardinal mixer for 30 min. Each variant had an average weight of 150 g. Aliquots of 50 g from each microencapsulated variant were analyzed immediately (I) or after daylight irradiation for 14 days at 25 °C (TCL), or after storage in vegetable capsules (C), for 14 days at 25 °C. The vegetal capsules had size 00 (provided from www.nutramax.ro, accessed on 10 July 2021) and the powders were introduced using an ALL-IN Capsule Filling Machine (Optics Technology, Delhi, India).

### 4.2. FTIR Analysis

The ATR-FTIR-MIR absorption spectra were recorded on a Shimadzu IR Prestige-21 spectrometer with a horizontal diamond accessory ATR (attenuated total reflectance) and a single reflection PIKE against hexane as background (control).

The spectra were recorded in the wavelength range 3600–650 cm^−1^ at a resolution of 4 cm^−1^, and 64 scans were collected for one spectrum. The primary data obtained were processed using IR solution Software Overview (Shimadzu, Kyoto, Japan) and OriginR 7SR1 Software (OriginLab Corporation, Northampton, MA, USA). The absorption bands characteristic of the different types of bonds and functional groups (expressed in cm^−1^) were identified. All measurements were done in triplicate.

According to the literature data, the different absorptions were especially followed [54,64]. Table 4 includes the most significant bands that correspond to specific vibrations of the molecules found in EOs.

From each FTIR spectrum, a matrix representing the peak intensities as a function of wavenumber (WN) was built. For data interpretation and statistical analysis, the mean values from each triplicate were considered.

### 4.3. Chemometrics

The raw FTIR data representing the absorption intensities at each wavenumber from the whole region (3600–650 cm^−1^) were statistically processed using the Unscrambler X10.4 software (Camo, Oslo, Norway). The multivariate unsupervised analysis principal component analysis (PCA) was applied first as a preliminary evaluation of the sample classification. In a second step, a semi-targeted statistical analysis was applied using the web-based software for metabolomics, Metaboanalyst 5.0 (https://www.metaboanalyst.ca/, accessed on 10 July 2021). One-way ANOVA analysis included the PLSDA score plots, correlation matrices and heatmap, and random forest analysis.

## 5. Conclusions

The characterization of essential oils and their derived formulations can be successfully done by ATR-FTIR-MIR spectrometry, having multiple advantages for both researchers in pharmaceutical and food sciences as well in the cosmetics industry. Microencapsulation of oils on fructose or maltodextrin matrices (powders, capsules, and chewable tablets) brings benefits related to improved stability.

Despite the complexity of the matrix, this method coupled with univariate or multivariate analysis shows high potential for global quantification of different encapsulated formulations. Beside saving time, this ATR-FTIR-MIR method can be easily used as a routine analysis in the EOs-related industry, as quality and safety control.

## Figures and Tables

**Table 1:** Wavenumbers WN (cm^−1^) and intensity values for the oily formulations Biomicin, Biomicin Forte, and Biomicin urinary, considering FTIR regions from 650 to 3600 cm^−1^.

Range (cm^−1^)	Biomicin		Biomicin Forte		Biomicin Urinary	
	WN (cm^−1^)	Intensity	WN (cm^−1^)	Intensity	WN (cm^−1^)	Intensity
650–1155 cm^−1^	723	0.567	721	0.581	719	0.638
	744	0.385	-	-	744	0.405
	796	0.326	-	-	785	0.203
	817	0.332	810	0.576	810	0.585
	848	0.265	-	-	-	-
	862	0.273	856	0.271	864	0.407
	887	0.339	891	0.223	887	0.257
	914	0.394	914	0.324	918	0.246
	948	0.337	947	0.356	941	0.344
	-	-	-	-	972	0.307
	995	0.411	993	0.307	993	0.432
	1033	0.481	1035	0.409	1031	0.279
	1070	0.399	1058	0.349	1058	0.34
	1097	0.534	1091	0.573	-	-
	1124	0.633	1120	0.582	1118	0.771
	1153	0.941	1151	0.947	1172	0.87
	1199	0.628	-	-	-	-
1200–1500 cm^−1^	1234	0.687	1230	0.718	1234	0.68
	1267	0.637	1267	0.615	1255	0.636
	-	-	1288	0.493	1269	0.445
	-	-	-	-	1301	0.377
	-	-	1363	0.339	1361	0.377
	1375	0.414	1377	0.35	1379	0.381
	-	-	1421	0.417	1421	0.529
	1462	0.560	1460	0.581	1458	0.627
	-	-	-	-	1502	0.149
1500–1800 cm^−1^	1514	0.458	1514	0.382	1519	0.161
	1608	0.125	1585	0.112	1591	0.226
	-	-	1616	0.138	1622	0.206
	1641	0.116	1639	0.081	1668	0.234
	-	-	-	-	1724	0.546
	1743	0.996	1743	0.795	1745	0.652
2800–3600 cm^−1^	2854	0.680	2854	0.644	2854	0.629
	2924	1	2924	1	2924	1
	2953	0.522	2956	0.544	2956	0.604
	3007	0.207	3007	0.181	3008	0.181

**Table 2:** Comparative recognition patterns of microencapsulated products on fructose and maltodextrin, based on wavenumbers (WNs) and modifications induced by light exposure (TCL) compared with initial (I) and capsules (C). The effects of the light irradiation were expressed in percentages of increases or decreases of absorption intensity (%TCL/I) on microencapsulated products in fructose (BF, BFF, BUF) or in maltodextrin (BM, BFM, BUM), measured by the ratios of intensities TCL/I, at different wavenumbers.

Microencapsulated Products	Fructose		Maltodextrin	
	WN I and C	WN TCL (% TCL/I)	WN I and C	WN TCL (% TCL/I)
Biomicin	746	-	813	-
	-	-	993	-
	-	-	1114	-
	-	-	1232	1232 (+14%)
	-	-	1265	1265 (+9.1%)
	-	-	-	1296 (0)
	1514	1512 (−4%)	1514	1514 (+10%)
	1604	1604 (−8%)	1606	1606 (+17%)
	1637	1637 (−11.6%)	-	-
	1745	1745 (0)	1766	1749 (+10%)
	-	-	2956	2956 (+8%)
Biomicin Forte	-	-	808	808 (−14.6%)
	-	-	900	-
	-	-	916	916 (0)
	-	-	993	-
	-	-	1114	1114 (−4.2%)
	-	-	1228	1228 (−5.1%)
	-	-	1263	1263 (−9.4%)
	-	-	1288	1288 (−5.1%)
	1514	1514 (0)	1512	1512 (−14.1%)
	-	-	1539	1539 (+27%)
	1614	-	1616	1616 (+13.8%)
	-	-	2956	2956 (-6.7%)
Biomicin urinary	748	750 (+17%)	-	-
			810	810 (−6.5%)
	-	-	993	993 (+0.8%)
	-	-	1112	1112 (−12.6%)
	-	-	1232	1232 (−0.8%)
	-	-	1253	-
	-	-	1379	1379 (+3%)
	1589	1589 (+29%)	1589	1589 (−4%)
	1622	1622 (+29%)	1622	1622 (−2.5%)
	1664	1664 (+20%)	-	-
			2956	2956 (−6.7%)

**Table 3:** The codes of different products obtained by microencapsulation of oily formulations Biomicin (B), Biomicin Forte (BF), and Biocin urinary (BU) on fructose (F) and maltodextrin (M). The codes for initial stage (I), after light irradiation (TCL), and in capsules (C) are also mentioned.

Sample Codes	I	TCL	C
Fructose	Fructose_I	Fructose_TCL	-
Biomicin in Fructose (BF)	BF_I	BF_TCL	CBF
Biomicin forte in Fructose (BFF)	BFF_I	BFF_TCL	CBFF
Biomicin urinary in Fructose (BUF)	BUF_I	BUF_TCL	CBUF
Maltodextrin	Maltodextrin_I	Maltodextrin_TCL	-
Biomicin in maltodextrin (BM)	BM_I	BM_TCL	CBM
Biomicin forte in maltodextrin (BFM)	BFM_I	BFM_TCL	CBFM
Biomicin urinary in maltodextrin (BUM)	BUM_I	BUM_TCL	CBUM

**Table 4:** Typical FTIR regions of volatile oils, correlated with the mode of vibration for specific functional groups, considered as predictor variables for statistical data processing.

Range (cm^−1^)	Functional Group	Mode of Vibration
3029–2989	=C–H (trans and cis)	Stretching
1745	-C=O	Stretching
1635–1650	RHC=CH_2_	Deformation
1375, 1450	=CH_2_	Deformation in plane
1380, 1458	-CH_2_^−^, dimethyl	Stretching; bending
1147–1159	-C–O, C-OH	Stretching, bending
850–920	=CH_2_	Out of plane deformation
842–862	Isopropyl group	Out of plane deformation
810	-C–H; -HC=CH^−^ (cis)	Bending (out of plane)

## Supplementary Materials

Table S1: FTIR maximal wavenumbers of the oily products Biomicin (Biomicin), compared with sunflower oil (SFO) used as solvent for the essential oils. Table S2: FTIR maximal wavenumbers of the oily products Biomicin (Biomicin forte), compared with sunflower oil (SFO) used as solvent for the essential oils. Table S3: FTIR maximal wavenumbers of the oily products Biomicin (Biomicin urinary), compared with sunflower oil (SFO) used as solvent for the essential oils. Table S4: FTIR intensity maxima in the wavenumber (WN) region 650–3500 cm^−1^ and specific of the powders (Biomicin (B)) obtained after incorporation in fructose (F), at initial stage (I), after light irradiation at room temperature (TCL), and kept in the dark, in gelatin capsules (C) encapsulation. Table S5: FTIR intensity maxima in the wavenumber (WN) region 650–3500 cm^−1^ and specific of the powders (Biomicin forte (BF)) obtained after incorporation in fructose (F), at initial stage (I), after light irradiation at room temperature (TCL), and kept in the dark, in gelatin capsules (C) encapsulation. Table S6: FTIR intensity maxima in the wavenumber (WN) region 650–3500 cm^−1^ and specific of the powders (Biomicin urinary (BU)) obtained after incorporation in fructose (F), at initial stage (I), after light irradiation at room temperature (TCL), and kept in the dark, in gelatin capsules (C) encapsulation. Table S7: FTIR intensity maxima in the wavenumber (WN) region 650–3500 cm^−1^ of the powders (Biomicin (B)) obtained after incorporation in maltodextrin (M), at initial stage (I), after light irradiation at room temperature (TCL), and kept in the dark, in gelatin capsules (C). Table S8: FTIR intensity maxima in the wavenumber (WN) region 650–3500 cm^−1^ of the powders (Biomicin forte (BF)) obtained after incorporation in maltodextrin (M), at initial stage (I), after light irradiation at room temperature (TCL), and kept in the dark, in gelatin capsules (C). Table S9: FTIR intensity maxima in the wavenumber (WN) region 650–3500 cm^−1^ of the powders (Biomicin urinary (BU)) obtained after incorporation in maltodextrin (M), at initial stage (I), after light irradiation at room temperature (TCL), and kept in the dark, in gelatin capsules (C). Figure S1: Superposed ATR-FTIR spectra (650–3500 cm^−1^) of fructose (a) and maltodextrin (b) at initial stage (I—dark line) and after daylight irradiation over 14 days at 25 °C (TCL—redline). Figure S2: The correlation maps between microencapsulated samples on fructose (a) and maltodextrin (b). For sample abbreviations, see Materials and Methods.
