# Supplementary material for: ATR-FTIR-MIR Spectrometry and Pattern Recognition of Bioactive Volatiles in Oily versus Microencapsulated Food Supplements: Authenticity, Quality, and Stability
Source: Molecules. 2021 Aug 10;26(16):4837. doi: 10.3390/molecules26164837 (PMC8401874; doi:10.3390/molecules26164837)
Supplement: Supplementary file 1 [file molecules-26-04837-s001.zip › Figure S1.pdf]

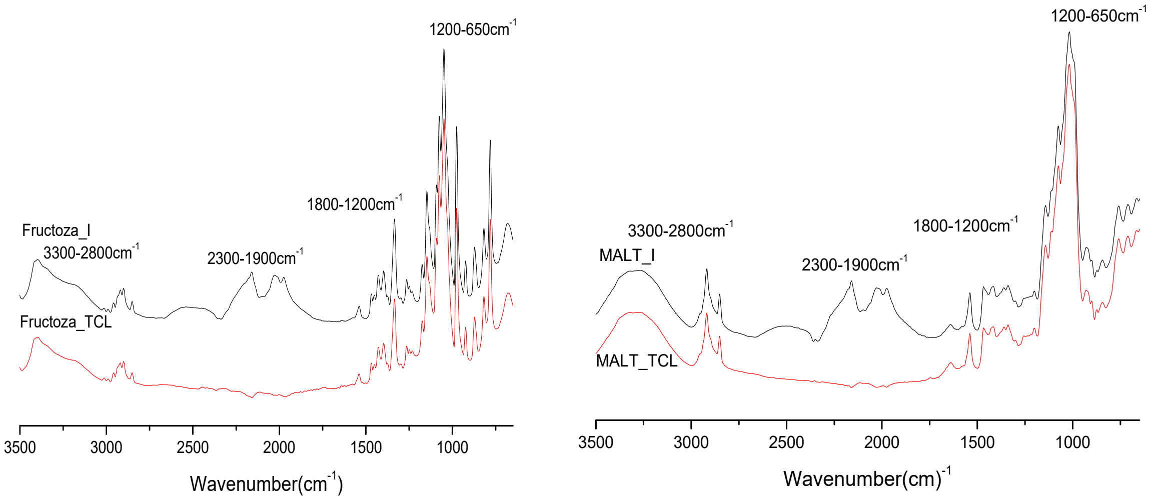

(**a**) (**b**)

Figure S1. Superposed ATR-FTIR spectra (650–3500 cm^{-1}) of fructose (**a**) and maltodextrin (**b**) at initial stage (I-dark line) and after daylight irradiation during 14 days at 25 °C (TCL redline).
