# Supplementary material for: ATR-FTIR-MIR Spectrometry and Pattern Recognition of Bioactive Volatiles in Oily versus Microencapsulated Food Supplements: Authenticity, Quality, and Stability
Source: Molecules. 2021 Aug 10;26(16):4837. doi: 10.3390/molecules26164837 (PMC8401874; doi:10.3390/molecules26164837)
Supplement: Supplementary file 1 [file molecules-26-04837-s001.zip › Figure S2.pdf]

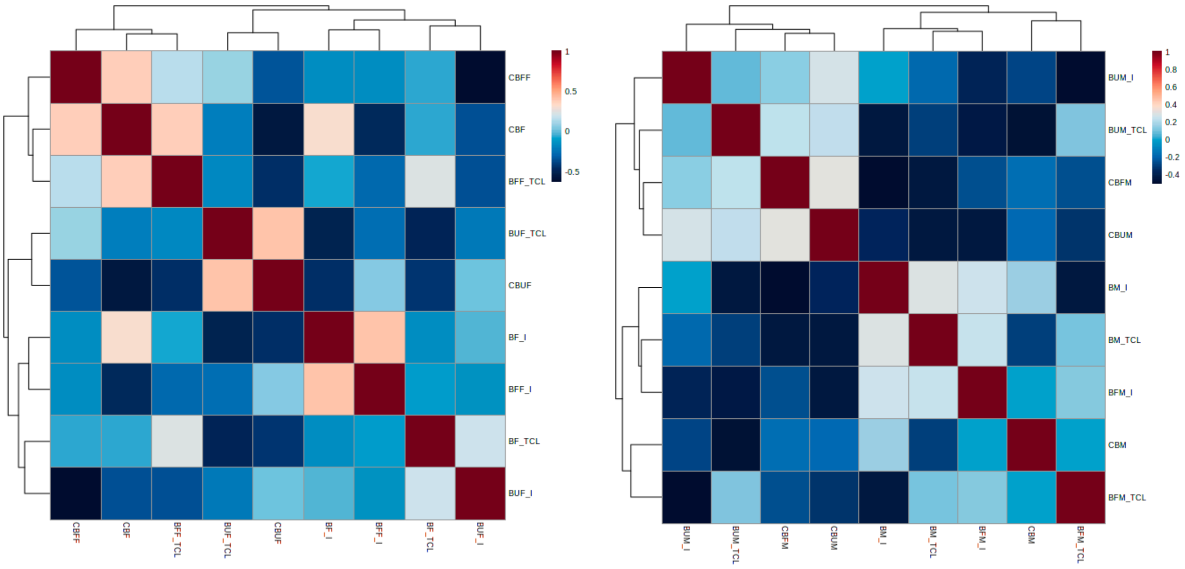

(**a**) (**b**)

Figure S2. Correlation maps between microencapsulated samples on fructose (**a**) and maltodextrin (**b**). For sample abbreviations see Materials and Methods.
