# Supplementary material for: ATR-FTIR-MIR Spectrometry and Pattern Recognition of Bioactive Volatiles in Oily versus Microencapsulated Food Supplements: Authenticity, Quality, and Stability
Source: Molecules. 2021 Aug 10;26(16):4837. doi: 10.3390/molecules26164837 (PMC8401874; doi:10.3390/molecules26164837)
Supplement: Supplementary file 1 [file molecules-26-04837-s001.zip › Tables S1-S3.pdf]

Table S1. FTIR maximal Wavenumbers of the oily products Biomicin (Biomicin (clove and tea tree in SFO), comparative to Sunflower oil (SFO) used as solvent for the essential oils.

Biomicin	SFO	Tea tree	Clove
723	721	727	721
744	-	781	744
794	-	798	792
817	-	823	817
848	846	864	850
889	871	887	-
912	912	923	912
948	966	948	947
995	-	997	995
1033	1031	1022	1031
1068, 1097	1097	1051, 1068	-
1122	1120	1126	1120,
1149	1159	1161	1147, 1199
1232	1236	1222	1232
1267	-	1249	1265
	-	1303	
1367	1375, 1398	1379	1367
1433	1419	1442	1431
1452, 1462	1462	1465	1462
1512	-	1512	1510
1606	-	1610	1606
1639	1653	1641,1656	1637
1743	1743	-	1762
-	-	**2725**	-
2854	2852	2875	2841
2924	2922	2916	2910, 2937
2958	-	2960	2962, 2972
3007	3007	-	3003

Table S2. FTIR maximal Wavenumbers of the oily products Biomicin (Biomicin forte (clove and thyme in SFO), comparative to Sunflower oil (SFO) used as solvent for the essential oils.

Biomicin forte	SFO	Thyme	Clove
721	721	719	721
**-**	-	736	744
**-**	-	783	792
813	-	810	817
850	846	858	850
-	871	887	-
912	912	921	912
947	966	945	947
993	-	995	995
1035	1031	1018	1031
1056	-	1056	-
1091	1097	1087	-

1122	1120	1111	1120
1149	1159	1151	1147
1184	-	1180	1199
1232	1236	1226	1232
1267	-	1261	1265
1367	-	1288	-
-	-	1338	-
-	-	1363	-
-	1375, 1398	1379	1367
-	1419	1419	1431
1431, 1460	1462	1456	1462
1512	-	1514	1510
-	-	1583	-
1610	-	1618	1606
1637	1653	1649	1637
1743	1743	1730	1762
2727	-	2729	-
-	-	2821	2841
2854	2852	2870	-
2924	2922	2927	2910, 2937
2956	-	2960	2962, 2972
3007	3007	3018	3003

Table S3. FTIR maximal Wavenumbers of the oily products Biomicin (Biomicin urinary (thyme, oregano, juniperus and cinnamon in SFO), comparative to Sunflower oil (SFO) used as solvent for the essential oils.

Biomicin urinary	SFO	Thyme	Oregano	Juniperus	Cinnamon
690		719	717		686
719	721	736	756	732	746
-	-	783	783	786	-
810	-	810	812	813	817
864	846	858	866	864	842
887	871	887	-	889	-
918	912	921	-	-	918
945, 972	966	945	939	954	972
993	-	995	993	989	1004
1031	-	1018	1033	1016	-
1058	1031	1056	1058	1064	-
-	1097	1087	-	1085	1072
1122	1120	1111	1116	1103	-
-	1159	1151	-	1124	1120
1174	-	1180	1172	1163, 1180, 1203	1157, 1178
1234	1236	1226	1232	1219	-
1255	-	1261	1251	1244, 1263	1236
1301	-	1288	1301	1303	1294
-	-	1338	1346	1330	1328
1361	-	1363	1361	1367	-

1379	1375	1379	1381	1379	1379
1421	1398, 1419	1419	1419	-	-
1458, 1502	1462	1456	1458, 1502	1442	1448, 1492
1519	-	1514	1519	1514	1512
1593	-	1583	1587	1595	1575, 1600
1624	-	1618	1620		1624
-	1653	1649	-	1649	1672
1743	1743	1730	1726	1737	1734
2854	2852	2870	2870	2875	2740, 2814
2924	2922	2927	2926	2918	2924
2956	-	2960	-	-	2962
