# Supplementary material for: ATR-FTIR-MIR Spectrometry and Pattern Recognition of Bioactive Volatiles in Oily versus Microencapsulated Food Supplements: Authenticity, Quality, and Stability
Source: Molecules. 2021 Aug 10;26(16):4837. doi: 10.3390/molecules26164837 (PMC8401874; doi:10.3390/molecules26164837)
Supplement: Supplementary file 1 [file molecules-26-04837-s001.zip › Tables S4-S6 Biomicin in Fructose .pdf]

Table S4. FTIR intensity maxima in the wavenumber (WN) region 650-3500 cm^{-1} and specific of the powders (Biomicin (B) incorporated in Fructose) obtained after incorporation in Fructose (F), at initial stage (I), after light irradiation at room temperature (TCL) and kept in dark, in gelatin capsules (C) encapsulation.

Range (cm^{-1})	**Fructose_I**		**BF_I**		**BF_TCL**		**CBF**	
	WN (cm^{-1})	Intensity	WN (cm^{-1})	Intensity	WN (cm^{-1})	Intensity	WN (cm^{-1})	Intensity
650-1200 cm^{-1}	680	0.374	677	0.416	671	0.396	678	0.425
	-	-	**746**	**0.309**	-	-	**748**	**0.315**
	781	0.674	781	0.711	781	0.671	781	0.747
	817	0.358	815	0.432	817	0.398	815	0.437
	871	0.289	869	0.343	871	0.314	869	0.357
	923	0.24	921	0.329	923	0.297	921	0.341
	975	0.722	974	0.743	975	0.725	974	0.778
	1049	1	1047	1	1047	1	1047	1
	1076	0.758	1074	0.792	1074	0.797	1074	0.789
	1091	0.512	1091	0.57	1091	0.583	1091	0.55
	1147	0.492	1147	0.542	1147	0.508	1145	0.547
	1174	0.228	1174	0.292	1174	0.278	1174	0.286
1200-1800 cm^{-1}	1232	0.131	1232	0.228	1232	0.186	1232	0.225
	1249	0.15	-	-	-	-	-	-
	1265	0.174	1265	0.268	1265	0.216	1265	0.278
	1298	0.108	1294	0.142	1296	0.13	1294	0.146
	1334	0.390	1334	0.398	1334	0.373	1334	0.424
	1375	0.115	1373	0.161	1373	0.14	1373	0.164
	1396	0.204	1396	0.224	1396	0.208	1396	0.233
	1427	0.189	1429	0.24	1427	0.207	1427	0.244
	1450	0.117	1452	0.164	1452	0.143	1450	0.163
	1467	0.123	1467	0.171	1467	0.152	1467	0.164
	-	-	**1514**	**0.149**	**1512**	**0.085**	**1514**	**0.143**
	1539	0.078	1539	0.101	1539	0.102	1539	0.081
	-	-	**1604**	**0.047**	**1604**	**0.045**	**1606**	**0.043**
	-	-	**1637**	**0.043**	**1637**	**0.039**	**1637**	**0.038**
	-	-	**1745**	**0.028**	**1745**	**0.026**	**1745**	**0.030**
1900-2300 cm^{-1}	1975	0.182	1975	0.148	-	-	-	-
	2029	0.189	2029	0.151	-	-	-	-
	2160	0.201	2160	0.163	-	-	-	-
2800-3300 cm^{-1}	2850	0.095	2850	0.12	2850	0.112	2850	0.106
	2900	0.142	2900	0.189	2900	0.162	2900	0.182
	2918	0.136	2918	0.178	2920	0.158	2920	0.161
	2958	0.09	2958	0.142	2958	0.111	2958	0.134
	3014	0.071	3012	0.087	3012	0.076	3012	0.078
	3396	0.245	3396	0.263	3396	0.249	3396	0.265

Table S5. FTIR intensity maxima in the wavenumber (WN) region 650-3500 cm^{-1} and specific of the powders (Biomicin forte (BF) incorporated in Fructose) obtained after incorporation in Fructose (F), at initial stage (I), after light irradiation at room temperature (TCL) and kept in dark, in gelatin capsules (C) encapsulation.

Range (cm^{-1})	Fructose_I		BFF_I		BFF_TCL		CBFF	
	WN (cm^{-1})	Intensity	WN (cm^{-1})	Intensity	WN (cm^{-1})	Intensity	WN (cm^{-1})	Intensity
650-1200 cm^{-1}	680	0.374	671	0.402	671	0.439	677	0.438
	781	0.674	781	0.667	781	0.703	781	0.718
	817	0.358	815	0.426	815	0.424	815	0.447
	871	0.289	869	0.32	869	0.341	869	0.359
	923	0.24	921	0.31	923	0.316	921	0.346
	975	0.722	974	0.707	974	0.744	974	0.772
	1049	1	1047	1	1047	1	1047	1
	1076	0.758	1074	0.809	1074	0.796	1076	0.786
1200-1800 cm^{-1}	1091	0.512	1091	0.604	1091	0.578	1091	0.586
	1147	0.492	1147	0.535	1147	0.526	1145	0.542
	1174	0.228	1174	0.298	1174	0.291	1174	0.287
	1232	0.131	1230	0.231	1230	0.199	1230	0.23
	1249	0.15	-	-	1249	0.196	-	-
	1265	0.174	1265	0.239	1265	0.226	1265	0.263
	1298	0.108	1290	0.153	1294	0.146	1292	0.176
	1334	0.39	1334	0.359	1334	0.396	1334	0.415
	1375	0.115	1375	0.143	1375	0.146	1375	0.175
	1396	0.204	1396	0.203	1396	0.22	1396	0.24
	1427	0.189	1427	0.234	1427	0.225	1427	0.258
	1450	0.117	1452	0.17	1452	0.152	1452	0.185
	1467	0.123	1467	0.175	1467	0.162	1467	0.184
	-	-	**1514**	**0.12**	**1514**	**0.08**	**1514**	**0.124**
	1539	0.078	1539	0.125	1539	0.109	1539	0.113
	-	-	1583	0.051	-	-	1583	0.06
	-	-	**1614**	**0.054**	**-**	**-**	**1614**	**0.062**
	-	-	-	-	-	-	**1745**	**0.041**
	1975	0.182	1975	0.133	-	-	-	-
	2029	0.189	2027	0.133	-	-	-	-
	2160	0.201	2160	0.144	-	-	-	-
2800-3300 cm^{-1}	2850	0.095	2850	0.127	2850	0.109	2852	0.129
	2900	0.142	2900	0.172	2900	0.161	2900	0.184
	2918	0.136	2920	0.192	2920	0.16	2920	0.188
	2958	0.09	2958	0.146	2958	0.12	2958	0.152
	3014	0.071	3012	0.077	3012	0.075	3012	0.088
	3396	0.245	3396	0.236	3398	0.254	3396	0.265

Table S6. FTIR intensity maxima in the wavenumber (WN) region 650-3500 cm^{-1} and specific of the powders (Biomicin urinary (BU).incorporated in Fructose) obtained after incorporation in Fructose (F), at initial stage (I), after light irradiation at room temperature (TCL) and kept in dark, in gelatin capsules (C) encapsulation.

Range (cm^{-1})	**Fructose_I**		**BUF_I**		**BUF_TCL**		**CBUF**	
	WN (cm^{-1})	Intensity	WN (cm^{-1})	Intensity	WN (cm^{-1})	Intensity	WN (cm^{-1})	Intensity
650-1200 cm^{-1}	680	0.374	682	0.379	669	0.432	669	0.412
	-	-	**748**	**0.273**	**750**	**0.335**	**748**	**0.321**
	781	0.674	781	0.636	781	0.669	781	0.591
	817	0.358	815	0.374	815	0.435	813	0.414
	871	0.289	869	0.311	869	0.359	869	0.327
	923	0.24	923	0.262	923	0.318	923	0.288
	975	0.722	974	0.717	974	0.735	974	0.675
	1049	1	1047	1	1047	1	1047	1
	1076	0.758	1074	0.735	1074	0.802	1074	0.801
	1091	0.512	1091	0.509	1091	0.602	-	-
	1147	0.492	1145	0.464	1145	0.519	1145	0.497
	1174	0.228	1174	0.25	1174	0.324	1174	0.314
1200-1800 cm^{-1}	1232	0.131	1232	0.169	1232	0.221	1230	0.218
	1249	0.15	1249	0.189	1249	0.237	1246	0.219
	1265	0.174	1261	0.189	1261	0.235	-	-
	1298	0.108	1300	0.123	1300	0.164	1300	0.141
	1334	0.39	1334	0.355	1334	0.391	1334	0.318
	1375	0.115	1375	0.124	1375	0.167	-	-
	1396	0.204	1396	0.195	1396	0.235	1396	0.188
	1427	0.189	1425	0.208	1425	0.248	1425	0.227
	1450	0.117	1452	0.141	1452	0.183	1454	0.171
	1467	0.123	1467	0.133	1467	0.182	-	-
	1539	0.078	1539	0.079	1539	0.123	1539	0.116
	-	-	**1589**	**0.051**	**1589**	**0.082**	**1587**	**0.066**
	-	-	**1622**	**0.054**	**1622**	**0.079**	**1622**	**0.069**
	-	-	**1664**	**0.067**	**1664**	**0.085**	**1664**	**0.081**
1900-2300 cm^{-1}	1975	0.182	1975	0.103	-	-	-	--
	2029	0.189	2029	0.105	-	-	-	-
	2160	0.201	2160	0.113	-	-	-	-
2800-3300 cm^{-1}	2850	0.095	2852	0.093	2852	0.131	2852	0.112
	2900	0.142	2900	0.148	2900	0.184	2900	0.152
	2918	0.136	2920	0.143	2920	0.187	2920	0.174
	2958	0.09	2958	0.133	2958	0.168	2958	0.142
	3014	0.071	3012	0.069	3014	0.095	3014	0.064
	3396	0.245	3396	0.226	3396	0.265	3394	0.213
