# Supplementary material for: ATR-FTIR-MIR Spectrometry and Pattern Recognition of Bioactive Volatiles in Oily versus Microencapsulated Food Supplements: Authenticity, Quality, and Stability
Source: Molecules. 2021 Aug 10;26(16):4837. doi: 10.3390/molecules26164837 (PMC8401874; doi:10.3390/molecules26164837)
Supplement: Supplementary file 1 [file molecules-26-04837-s001.zip › Tables S7-S9 Biomicin in Maltodextrin .pdf]

Table S7. FTIR intensity maxima in the wavenumber (WN) region 650-3500 cm^{-1} of the powders (Biomicin (B) incorporated in Maltodextrin) obtained after incorporation in Maltodextrin (M), at initial stage (I), after light irradiation at room temperature (TCL) and kept in dark, in gelatin capsules (C).

Range (cm^{-1})	**Maltodextrin_I**		**BM_I**		**BM_TCL**		**CBM**	
	WN (cm^{-1})	Intensity	WN (cm^{-1})	Intensity	WN (cm^{-1})	Intensity	WN (cm^{-1})	Intensity
650-1200 cm^{-1}	663	0.484	665	0.484	665	0.484	-	-
	**709**	**0.467**	709	0.475	709	0.472	711	0.469
	758	0.462	756	0.481	758	0.47	754	0.471
	-	-	**813**	**0.294**	**-**	**-**	**813**	**0.321**
	844	0.295	848	0.286	846	0.28	848	0.308
	873	0.262	873	0.283	873	0.267	-	-
	-	-	916	0.367	918	0.347	918	0.386
	-	-	**993**	**0.93**	**-**	**-**	**995**	**0.947**
	1018	1	1018	1	1016	1	1018	1
	1076	0.709	1072	0.656	1074	0.649	1074	0.67
	-	-	**1114**	**0.503**	**-**	**-**	**1114**	**0.538**
	1143	0.463	1143	0.461	1143	0.443	1145	0.494
1200-1800 cm^{-1}	1201	0.199	1201	0.245	1201	0.219	1201	0.281
	-	-	**1232**	**0.241**	**1232**	**0.207**	**1232**	**0.275**
	-	-	**1265**	**0.251**	**1265**	**0.208**	**1265**	**0.274**
	-	-	-	-	**1296**	**0.161**	-	-
	1338	0.216	1338	0.214	1338	0.203	1338	0.219
	1361	0.209	1363	0.238	1363	0.214	1365	0.249
	1415	0.213	1429	0.248	1429	0.218	1429	0.256
	1467	0.212	1465	0.22	1465	0.204	1463	0.239
	-	-	**1514**	**0.192**	**1514**	**0.141**	**1514**	**0.212**
	1539	0.194	1539	0.145	1539	0.15	1539	0.159
	-	-	**1606**	**0.08**	**1608**	**0.071**	**1606**	**0.094**
	1639	0.095	1637	0.09	1637	0.084	1637	0.103
	-	-	**1766**	**0.051**	**1749**	**0.033**	**1751**	**0.056**
1900-2300 cm^{-1}	1975	0.208	1975	0.16	-	-	-	-
	2027	0.209	2027	0.164	-	-	-	-
	2160	0.231	2160	0.178	-	-	-	-
2800-3300 cm^{-1}	2850	0.19	2850	0.17	2850	0.162	2850	0.176
	2918	0.268	2918	0.259	2918	0.243	2918	0.262
	-	-	**2956**	**0.164**	**-**	**-**	**2956**	**0.174**
	3265	0.263	3267	0.243	3267	0.233	3323	0.24

Table S8. FTIR intensity maxima in the wavenumber (WN) region 650-3500 cm^{-1} of the powders (Biomicin forte (BF) incorporated in Maltodextrin) obtained after incorporation in Maltodextrin (M), at initial stage (I), after light irradiation at room temperature (TCL) and kept in dark, in gelatin capsules (C).

Domeniu (cm^{-1})	**Maltodextrin_I**		**BFM_I**		**BFM_TCL**		**CBFM**	
	WN (cm^{-1})	Intensity	WN (cm^{-1})	Intensity	WN (cm^{-1})	Intensity	WN (cm^{-1})	Intensity
650-1200 cm^{-1}	663	0.484	665	0.514	665	0.527	665	0.513
	709	0.467	711	0.5	709	0.508	715	0.495
	758	0.462	756	0.502	756	0.499	752	0.493

	-	-	**808**	**0.416**	**806**	**0.384**	**810**	**0.355**
	844	0.295	852	0.314	850	0.322	862	0.315
	873	0.262	873	0.293	871	0.295	-	-
	-	-	**900**	**0.329**	**-**	**-**	**900**	**0.299**
	-	-	**916**	**0.373**	**918**	**0.372**	**-**	**-**
	929	0.333	943	0.365	941	0.362	935	0.367
	-	-	**993**	**0.92**	-	-	**993**	**0.987**
	1018	1	1018	1	1016	1	1016	1
	1076	0.709	1076	0.689	1076	0.678	1074	0.668
	-	-	**1114**	**0.561**	**1112**	**0.537**	**1112**	**0.556**
	1143	0.463	1145	0.524	1145	0.497	1138	0.471
1200-1800 cm^{-1}	1201	0.199	1201	0.267	1201	0.260	1202	0.209
	-	-	**1228**	**0.292**	**1228**	**0.267**	**1228**	**0.208**
	-	-	**1263**	**0.276**	**1263**	**0.25**	**1253**	**0.246**
	-	-	**1288**	**0.235**	**1290**	**0.223**	**1300**	**0.191**
	1338	0.216	1338	0.238	1338	0.24	1340	0.221
	1361	0.209	1363	0.249	1361	0.244	1361	0.231
	1415	0.213	1421	0.297	1421	0.278	1379	0.213
	1467	0.212	1458	0.258	1465	0.253	1419	0.276
			**1512**	**0.198**	**1514**	**0.17**	**1458**	**0.238**
	-	-	**1539**	**0.162**	**1539**	**0.206**	**1539**	**0.154**
	1539	0.194	1581	0.091	1583	0.104	1589	0.099
	-	-	**1616**	**0.101**	**1618**	**0.115**	**1622**	**0.111**
	1639	0.095	1637	0.091	1637	0.115	1664	0.125
1900-2300 cm^{-1}	1975	0.208	1975	0.132	-	-	-	-
	2027	0.209	2029	0.135	-	-	-	-
	2160	0.231	2160	0.147	-	-	-	-
2800-3300 cm^{-1}	2850	0.19	2852	0.172	2850	0.192	2852	0.159
	2918	0.268	2918	0.271	2918	0.279	2920	0.249
	-	-	**2956**	**0.193**	**2956**	**0.18**	**2956**	**0.181**
	3265	0.263	3323	0.261	3321	0.26	3317	0.249

Table S9. FTIR intensity maxima in the wavenumber (WN) region 650-3500 cm^{-1} of the powders (Biomicin urinary (BU) incorporated in Maltodextrin) obtained after incorporation in Maltodextrin (M), at initial stage (I), after light irradiation at room temperature (TCL) and kept in dark, in gelatin capsules (C).

Range (cm^{-1})	**Maltodextrin_I**		**BUM_I**		**BUM_TCL**		**CBUM**	
	WN (cm^{-1})	Intensity	WN (cm^{-1})	Intensity	WN (cm^{-1})	Intensity	WN (cm^{-1})	Intensity
650-1200 cm^{-1}	663	0.484	665	0.494	663	0.518	665	0.499
	709	0.467	715	0.486	715	0.504	715	0.486
	758	0.462	750	0.495	752	0.501	750	0.481
	-	-	**810**	**0.367**	**810**	**0.343**	**810**	**0.356**
	873	0.262	860	0.309	860	0.307	860	0.315
	900	0.294	900	0.289	900	0.297	900	0.295
	929	0.333	937	0.359	935	0.355	935	0.373
	**-**	**-**	**993**	**0.966**	**993**	**0.974**	**993**	**1**
	1018	1	1018	1	1016	1	1016	0.999
	1076	0.709	1074	0.712	1074	0.646	1074	0.659

	-	-	**1112**	**0.602**	**1112**	**0.526**	**1112**	**0.545**
	1143	0.463	1136	0.496	1139	0.447	1139	0.462
1200-1800 cm^{-1}	1201	0.199	1199	0.225	1199	0.202	1199	0.207
	-	-	**1232**	**0.24**	**1253**	**0.234**	**1232**	**0.231**
	-	-	**1253**	**0.252**	**-**	**-**	**1253**	**0.241**
	-	-	**1301**	**0.185**	**1301**	**0.188**	**1301**	**0.19**
	1338	0.216	1340	0.216	1340	0.221	1340	0.217
	1361	0.209	1361	0.227	1361	0.233	1361	0.228
	-	-	**1379**	**0.209**	**1379**	**0.216**	**1379**	**0.211**
	1415	0.213	1419	0.283	1419	0.269	1419	0.271
	1467	0.212	1458	0.244	1458	0.234	1458	0.234
	1539	0.194	1539	0.158	1539	0.171	1539	0.147
	-	-	**1589**	**0.104**	**1580**	**0.1**	**1589**	**0.099**
	-	-	**1622**	**0.119**	**1622**	**0.116**	**1622**	**0.112**
	1639	0.095	1662	0.136	1662	0.124	1662	0.124
1900-2300 cm^{-1}	1975	0.208	1975	0.131	-	-	-	-
	2027	0.209	2025	0.135	-	-	-	-
	2160	0.231	2160	0.148	-	-	-	-
2800-3300 cm^{-1}	2850	0.19	2850	0.164	2852	0.169	2852	0.153
	2918	0.268	2918	0.257	2920	0.253	2920	0.238
	-	-	**2956**	**0.192**	**2956**	**0.179**	**2956**	**0.176**
	3265	0.263	3323	0.247	3273	0.257	3327	0.242
